# Fast Intracortical Sensory-Motor Integration: A Window Into the Pathophysiology of Parkinson’s Disease

**DOI:** 10.3389/fnhum.2019.00111

**Published:** 2019-04-08

**Authors:** Raffaele Dubbioso, Fiore Manganelli, Hartwig Roman Siebner, Vincenzo Di Lazzaro

**Affiliations:** ^1^Department of Neurosciences, Reproductive Sciences and Odontostomatology, University Federico II of Naples, Napoli, Italy; ^2^Danish Research Centre for Magnetic Resonance, Centre for Functional and Diagnostic Imaging and Research, Copenhagen University Hospital Hvidovre, Hvidovre, Denmark; ^3^Department of Neurology, Copenhagen University Hospital Bispebjerg, Copenhagen, Denmark; ^4^Institute for Clinical Medicine, Faculty of Health and Medical Sciences, University of Copenhagen, Copenhagen, Denmark; ^5^Unit of Neurology, Neurophysiology, Neurobiology, Department of Medicine, University Campus Bio-Medico, Rome, Italy

**Keywords:** short-latency afferent inhibition, cholinergic neuromodulation, cortical oscillations, dopaminergic dysfunction, Parkinson’s disease, movement disorder, neurophysiological biomarker

## Abstract

Parkinson’s Disease (PD) is a prototypical basal ganglia disorder. Nigrostriatal dopaminergic denervation leads to progressive dysfunction of the cortico-basal ganglia-thalamo-cortical sensorimotor loops, causing the classical motor symptoms. Although the basal ganglia do not receive direct sensory input, they are important for sensorimotor integration. Therefore, the basal ganglia dysfunction in PD may profoundly affect sensory-motor interaction in the cortex. Cortical sensorimotor integration can be probed with transcranial magnetic stimulation (TMS) using a well-established conditioning-test paradigm, called short-latency afferent inhibition (SAI). SAI probes the fast-inhibitory effect of a conditioning peripheral electrical stimulus on the motor response evoked by a TMS test pulse given to the contralateral primary motor cortex (M1). Since SAI occurs at latencies that match the peaks of early cortical somatosensory potentials, the cortical circuitry generating SAI may play an important role in rapid online adjustments of cortical motor output to changes in somatosensory inputs. Here we review the existing studies that have used SAI to examine how PD affects fast cortical sensory-motor integration. Studies of SAI in PD have yielded variable results, showing reduced, normal or even enhanced levels of SAI. This variability may be attributed to the fact that the strength of SAI is influenced by several factors, such as differences in dopaminergic treatment or the clinical phenotype of PD. Inter-individual differences in the expression of SAI has been shown to scale with individual motor impairment as revealed by UPDRS motor score and thus, may reflect the magnitude of dopaminergic neurodegeneration. The magnitude of SAI has also been linked to cognitive dysfunction, and it has been suggested that SAI also reflects cholinergic denervation at the cortical level. Together, the results indicate that SAI is a useful marker of disease-related alterations in fast cortical sensory-motor integration driven by subcortical changes in the dopaminergic and cholinergic system. Since a multitude of neurobiological factors contribute to the magnitude of inhibition, any mechanistic interpretation of SAI changes in PD needs to consider the group characteristics in terms of phenotypical spectrum, disease stage, and medication.

## Introduction

Parkinson’s disease (PD) is a neurodegenerative disorder affecting multiple neuromodulatory transmitter systems (Barone, 2010). The cardinal motor symptoms of PD are due to the progressive loss of nigrostriatal dopaminergic neurons in the midbrain. Progressive nigrostriatal dopaminergic denervation causes a dysfunction in the cortex-basal ganglia sensorimotor loops, producing slowness of movements, rigidity, tremor, and difficulties with gait and balance (Dickson, 2012). Although the basal ganglia do not receive direct somatosensory input from the periphery, several lines of evidence support the idea that the basal ganglia are important for gating sensory input for motor control through cortico-basal ganglia-thalamo-cortical re-entry loops (Haber and Calzavara, 2009). Specifically, primary and secondary somatosensory cortices in the parietal lobe send inputs to the striatum of the basal ganglia, where sensory cortical projections are topographically mapped (Künzle, 1977; Di Martino et al., 2008). The notion that the basal ganglia are relevant to **sensorimotor integration** is rather old. Back in 1985, Lidsky introduced the notion that the basal ganglia serve as “sensory analyzer for motor systems” which “ultimately affect movement by gating sensory inputs into other motor areas” (Lidsky et al., 1985). Lesions of the basal ganglia mostly affect automatic movements that need sensory guidance, pointing towards a role of the basal ganglia in sensory-motor control of automatic or highly trained movements (Boecker et al., 1999).

KEY CONCEPT 1 **Sensorimotor integration**

Sensorimotor integration is the process whereby somatosensory input is integrated by the central nervous system to shape motor program execution. Parkinson’s disease (PD) is considered a pathological model of aberrant sensory-motor integration, where movement accuracy and speed are severely affected by the altered sensory feedback.

In addition, PD patients also exhibit impairment of selecting the appropriate response while simultaneously suppressing inappropriate response tendencies (Praamstra and Plat, 2001). Interestingly, patients with PD display difficulties in suppressing automatic response activation while proactive inhibitory control appears to be intact (Praamstra and Plat, 2001; Seiss and Praamstra, 2004; Wylie et al., 2009). Using transcranial magnetic stimulation (TMS), it was also shown that impaired inhibition also manifests itself within the corticomotor output system in PD (Kleine et al., 2001). The TMS-evoked excitation of the corticomotor projections produced an increased, prolonged and less synchronized excitation of the target muscle (Kleine et al., 2001). This converging evidence shows that anatomical and functional impairment of the cortico-basal ganglia-thalamo-cortical loop in PD profoundly affects sensory-motor integration in the cortex.

Sensorimotor integration at the cortical level can be probed non-invasively by pairing electrical stimulation of peripheral somatosensory afferents with focal TMS targeting the contralateral primary motor cortex (M1). In their seminal study, Tokimura et al. (2000) demonstrated that peripheral nerve stimulation at the contralateral wrist reduced the amplitude of motor evoked potentials (MEPs) when the TMS pulse was given to the primary motor hand area (M1-HAND) 2–8 ms after the arrival of the afferent volley in cortex. The term “**short-latency afferent inhibition**” (SAI) was coined for this conditioning-test paradigm, and SAI soon became a well-established neurophysiological technique to probe rapid intracortical sensorimotor integration in health and disease (Turco et al., 2018b). The inhibitory effect of the sensory input on the motor output provides a neurophysiological signature of fast sensory-motor integration. Three components constitute the SAI circuit that enables fast-integrative processing: the fast afferent-sensory pathway, the motor-efferent pathway and the integrative component in the sensorimotor cortex. The neuropharmacological profile of SAI is complex. The sensory input exerts its inhibitory effects on the corticospinal neurons through γ-Aminobutyric acid (GABA)-ergic intracortical circuits (Di Lazzaro et al., 2005c; Di Lazzaro and Ziemann, 2013), but its magnitude is also modulated by dopaminergic (Sailer et al., 2003) and cholinergic neuromodulatory circuits (Di Lazzaro et al., 2000). The fact that the magnitude of SAI is modulated by cholinergic drugs has provoked considerable interest in the use of SAI in patients with dementia. In patients with Alzheimer Disease (AD), a loss of SAI has been interpreted as an indicator of cortical cholinergic denervation and a normal SAI as a predictor of a positive effect of cholinergic medication on cognitive deficits (Di Lazzaro et al., 2000; Cantone et al., 2014).

KEY CONCEPT 2 **Short-latency afferent inhibition (SAI)**

Fast component of sensorimotor integration can be studied *in vivo* by examining the effects of sensory input on the motor output at the cortical level. The amplitude of a motor evoked potential (MEP) induced by transcranial magnetic simulation (TMS) over the motor cortex is reduced by a peripheral nerve stimulation few milliseconds before the TMS pulse. The magnitude of this inhibition represents the neurophysiological correlate of sensorimotor integration efficiency.

The neurophysiological and neuropharmacological properties of SAI have motivated researchers to use SAI as a tool to examine whether and how PD is associated with an impairment of fast sensory-motor integration in the pericentral sensorimotor cortex. In the same vein, researchers have examined whether the attenuation of SAI in individual patients scales with dopaminergic and cholinergic cortical neurodegeneration and is associated with particular clinical symptoms (Martin-Rodriguez and Mir, 2018). In this review, we first summarize some key features of SAI in the healthy human brain. We will then review the evidence for an alteration of SAI in PD and discuss, based on the published data, whether the individual reduction in SAI can be used as an electrophysiological biomarker of cholinergic or dopaminergic denervation of the sensorimotor cortex in PD. Finally, we will ask the question whether a reduction in SAI is associated with specific clinical manifestations of PD.

## Short-Latency Afferent Inhibition in the Healthy Human Brain

### Cortical Origin of SAI

Converging evidence supports the hypothesis that SAI is generated in the sensorimotor cortex, although the exact anatomic circuits generating SAI are still unknown. The most direct evidence that peripheral somatosensory input modulates the TMS-induced motor output at the cortical level comes from invasive recordings of corticospinal volleys in patients with implanted electrodes in the cervical epidural space (Tokimura et al., 2000). These studies showed that later I-waves (I2 and I3 waves) were reduced at an interval appropriate for SAI, whereas the early I-wave (I1 wave) remained unchanged. Based on these findings, it has been proposed that peripheral nerve stimulation activates glutamatergic thalamocortical projections onto intracortical GABA_A_-ergic interneurons which in turn, suppress the intracortical inhibitory GABA_A_-ergic circuits generating the late descending volleys (late I-waves) in the corticospinal tract (Di Lazzaro and Ziemann, 2013). A critical role of thalamocortical projections is substantiated by lesion studies, showing a marked reduction or loss of SAI in patients with unilateral (Oliviero et al., 2005) or bilateral (Nardone et al., 2010) paramedian thalamic stroke.

### Which Factors Modulate the Expression of SAI?

The relative strength of SAI depends on the magnitude of the sensory afferent input evoked by peripheral stimulation. The greater the afferent volley evoked by peripheral stimulation, the stronger is the magnitude of SAI (Bailey et al., 2016). The expression of SAI also depends on the somatotopic relation between the sensory input and motor output. Electrical stimulation of digits close to the TMS-target muscle (i.e., homotopic stimulation) induces stronger inhibition than stimulation of digits distant to the TMS-target muscle (i.e., heterotopic stimulation; Classen et al., 2000). The somatotopic organization of SAI was studied in detail using a neuronavigated TMS mapping technique which adjusts the coil position and orientation to the individual shape of the central sulcus (Dubbioso et al., 2017c). Mapping the input-output relationship of SAI revealed a center-surround organization in the human M1-HAND. SAI was evoked by homotopic stimulation only, whereas the conditioning effect produced the opposite effect, namely short-latency afferent facilitation (SAF), in the case of heterotopic stimulation (Dubbioso et al., 2017c).

The expression of homotopic is highly state dependent. In healthy individuals, SAI is consistently expressed at rest, but attenuated during finger movements (Dubbioso et al., 2017c). In the active target muscle, SAI was reduced at movement initiation during both mixed and homotopic cutaneous nerve stimulation (Asmussen et al., 2013; Cho et al., 2016), whereas SAI was reduced during the maintenance phase of the movement (Asmussen et al., 2013) or found to be normal (Cho et al., 2016). Accordingly, SAI and SAF by homotopic or heterotopic stimulation were abolished during the tonic contraction of the target muscle (Dubbioso et al., 2017c). This state-dependent pattern of SAI modulation can be attributed to a sensorimotor gating mechanism, which attenuates the perceived intensity of stimuli generated by movements. Although SAI is closely modulated by movement, no relationship between SAI magnitude and manual dexterity has been found (Turco et al., 2018c).

The expression of SAI is not only modulated by the intrinsic sensorimotor state but also shaped by transcranial brain stimulation. Transcranial alternating current stimulation (TACS) at 20 Hz completely abolished SAI in the relaxed muscle (Guerra et al., 2016). The suppressive effect of 20 Hz TACS on SAI did not depend on the phase relationship between TACS and the timing of the TMS pulse probing SAI (Guerra et al., 2016). This finding suggests a link between SAI expression and the oscillatory state of the sensorimotor cortex, yet it remains to be shown that SAI is also suppressed by physiologically generated beta oscillations in sensorimotor cortex.

The magnitude of SAI can also be modulated by TMS interventions, for instance when electrical stimulation of the median nerve is consistently paired with TMS of the contralateral M1-HAND at an inter-stimulus interval (ISI) of 25 ms (Quartarone et al., 2006) or with TMS over the contralateral S1 at an ISI of N20-2.5 ms (Tsang et al., 2015). Sub-motor threshold 5 Hz repetitive paired associative stimulation produced a long-lasting increase in corticospinal excitability along with an attenuation of SAI (Quartarone et al., 2006; Tsang et al., 2015). Interventional TMS protocols which are thought to induce homosynaptic plasticity, such as continuous theta burst (cTBS) have also been sued to modify SAI. While cTBS over M1-HAND failed to modulate SAI, cTBS delivered over S1 reduced SAI along with an increase in cortico-spinal excitability (Tsang et al., 2014).

Cognitive processes, for instance attention and working memory, shape afferent sensory-motor integration involving distinct intracortical circuits as demonstrated by single monophasic TMS pulses that evoke different current directions in the brain (Mirdamadi et al., 2017; Suzuki and Meehan, 2018). Specifically, it has been demonstrated that SAI evoked using antero-posterior (AP), but not posterior-anterior (PA), current is reduced by a concurrent visual detection task with high attention demands. These results suggested that only AP-elicited intracortical circuits are sensitive to cross-modal attention task by altering sensory processing in premotor areas (Mirdamadi et al., 2017). Instead, a verbal working memory task modulated SAI, regardless of the TMS-induced current direction in the brain (AP or PA), reflecting a generalized effect of this cognitive task across anatomically distinct circuits upon cortico-spinal neurons in the M1-HAND (Suzuki and Meehan, 2018).

The fact that intrinsically and extrinsically induced state changes in the sensorimotor system can dynamically tune the expression of SAI needs to be born in mind when SAI is considered as “biomarker” in PD.

### Influence of Neurotransmitter Systems on SAI Magnitude

Pharmacological and clinical studies provided converging evidence that the expression of SAI is modulated by several neurotransmitters such as acetylcholine, dopamine, GABA and noradrenaline (Turco et al., 2018b). SAI is significantly reduced by scopolamine, a muscarinic cholinergic antagonist, in young healthy adults (Di Lazzaro et al., 2000) and can be improved with rivastigmine, an acetylcholinesterase inhibitor, in patients with abnormal reduction of SAI, such as AD (Di Lazzaro et al., 2002). Cholinergic inhibition of pyramidal neurons has been demonstrated directly in experimental studies (Gulledge and Stuart, 2005). Interestingly, this rivastigmine effect on SAI predicted the long term response to cholinesterase inhibitor in patients with AD (Di Lazzaro et al., 2005a). The effects of scopolamine and rivastigmine suggest that SAI may be useful to probe *in vivo* the functional integrity of central cholinergic circuits of the human brain. These studies indicate that SAI can trace the functional impairment of central cholinergic circuits, allowing to discriminate for example cholinergic from non-cholinergic form of dementia (Di Lazzaro et al., 2006; Manganelli et al., 2014; Dubbioso et al., 2017b).

Beyond cholinergic transmission, the dopaminergic system plays a relevant role in the modulation of SAI, in accordance with a strong synaptic interaction between dopamine an acetylcholine signaling in different brain areas (Di Cara et al., 2007; Millan et al., 2007). L-dopa treatment has been shown to normalize SAI in patients with restless legs syndrome (Rizzo et al., 2010) and AD (Martorana et al., 2009; Nardone et al., 2014). **Dopaminergic medication** also influences SAI in patients with PD (for more details see section on PD).

KEY CONCEPT 3 **Dopaminergic medication and sensory processing**

Studies on PD patients consistently found reduced levels of SAI in the ON-medication state suggesting a role of dopamine replacement in driving this abnormality. Indeed, dopaminergic medication could lead to decreases in central processing or integration of sensory signals in PD patients. For instance, it has been shown that dopaminergic medication could worsen SAI and proprioception. The positive relationship between motor symptoms and SAI suppression in the ON-medication state suggests that the effect of medication may be more detrimental to SAI in patients that are less responsive to dopaminergic pharmacotherapy, for instance, patients with more prominent cholinergic involvement.

Regarding GABAergic system, in human cortical slices, it was observed that acetylcholine activated GABA neurons and triggered GABAergic postsynaptic currents (Alkondon et al., 2000). Thus, SAI may also be mediated through the interactions between cholinergic projections and specific GABAergic interneurons. This also explains the findings that the administration of positive GABA_A_ but not GABA_B_ receptor modulators influences SAI (Turco et al., 2018a). Zolpidem, a selective agonist of alpha1 subunit of GABA_A_ receptor, and lorazepam, a positive allosteric modulator of GABA_A_ receptor, significantly reduced SAI (Di Lazzaro et al., 2005b,c, 2007; Turco et al., 2018a), whereas diazepam, a non-selective agonist, induced a slight increase or no effect on SAI (Di Lazzaro et al., 2005c, 2007). This observation is presumably explained by a differential role of the different alpha subunits of GABA_A_ receptor in the modulation of afferent inhibition with a suppression of cholinergic inhibition by alpha1 subunit activation.

Lastly, a recent study has also demonstrated that acute and chronic intake of reboxetine, a noradrenaline reuptake inhibitor, reduces SAI, likely through suppression of GABAergic neurotransmission (Kuo et al., 2017).

## Fast Sensory Input and Motor Output Pathways in PD

As stated above the basal ganglia do not receive direct sensory input, yet patients with PD often report sensory symptoms (Pallis, 1971; Snider et al., 1976; Hillen and Sage, 1996). Objective somatosensory deficits are well documented in PD and have been mainly found in tasks that require the use of kinaesthetic sense such as conscious perception of limb position and motion in space proprioception and kinaesthesia (Schneider et al., 1987; Klockgether et al., 1995; Demirci et al., 1997; Jobst et al., 1997; Zia et al., 2000) or temporal or spatial discrimination (Conte et al., 2013).

The fast-afferent sensory volley eliciting SAI can be studied by recording the somatosensory evoked potentials (SSEPs) evoked by stimulation of the peripheral nerve. Most SSEP studies in PD have employed electrical stimulation of a mixed nerve that reflects activation of proprioceptive as well as cutaneous inputs. SSEP studies in patients with PD found a reduced amplitude of the late N30 component of the SSEP, while the early N20-P25 components were found to be normal (Rossini et al., 1989; Cheron et al., 1994; Ulivelli et al., 1999). SSEP studies following proprioceptive stimulation during passive flexion (Mima et al., 1996) or electric stimulation (Restuccia et al., 1999) of the proximal interphalangeal joint of the finger demonstrated that the origin of the N30 waveform is more complex than the early components, containing information from cutaneous afferents as well as from joint and tendinous inputs. Therefore, it has been hypothesized that the defective proprioception described in PD might be related to the depression of the N30 component.

The early cortical components of the SSEP, namely the dipole N20/P20 and P25 component, reflect early sensory processing in the pericentral cortex and are thought to give rise to fast sensory afferent inhibition. Since these early components are intact in PD patients, alterations of SAI in PD patients cannot be attributed to a dysfunction of the afferent sensory pathway. The same consideration applies to the fast cortico-motor output pathway which is unaffected in PD. Indeed, a TMS study, which recorded MEPs at increasing stimulus intensities, demonstrated a normal gain function of corticospinal excitability in PD patients (Kojovic et al., 2012).

However, context-dependent modulation of early cortical sensory processing is impaired in PD. Normal movement-related attenuation of perceived stimuli, referred to as sensorimotor gating, is deficient in patients with PD while they are off dopaminergic treatment and can be restored by dopamine replacement therapy (Macerollo et al., 2016). In contrast to healthy controls, the early N20-P25 SSEP components were not modulated at all by movement in patients with PD. The authors speculated that abnormalities in sensory gating may contribute to the difficulties in movement initiation observed in PD (Macerollo et al., 2016). Another study on PD patients treated with deep brain stimulation (DBS) of the subthalamic nucleus or globus pallidum found that movement of the hand ipsilateral to median nerve stimulation gated the subcortical triphasic negative–positive–negative potentials—at latencies of 14–18–22.5 ms, similar to cortical gating observed with SSEP at N20, P20, and N30 (Insola et al., 2004). Converging evidence suggests that sensory gating preceding the onset of movement seems to be mediated by motor cortical areas that contribute to preparation and execution of movement (Cohen and Starr, 1987; Seki and Fetz, 2012; Macerollo et al., 2018).

## Cortical Sensorimotor Integration in PD

Based on the work summarized in the previous sections, it can be concluded that SAI is a cortical process, albeit the exact circuit underlying this fast sensory-motor integration is still unknown. In addition, the lack of major impairment in the fast afferent sensory-to-cortical and efferent cortical-to-motor pathways in PD suggest that abnormalities of SAI in PD are caused by a dysfunction in fast intracortical sensorimotor integration.

Patients with electrodes implanted for DBS provide a unique opportunity to study the interplay between the subcortical target site and the cortex. Two electrophysiological studies showed that continuous high-frequency DBS of the STN modifies SAI in PD, confirming a close relationship between fast sensory-motor cortical integration and the basal ganglia. A first study examined medicated patients with the STN-DBS switched on or off (Sailer et al., 2007). SAI was reduced in the off-stimulation and was acutely restored after STN stimulation was resumed, suggesting that STN stimulation might normalize pathways that are adversely affected by dopaminergic medications (Sailer et al., 2007).

A second study focused on the long-term effect of STN-DBS on SAI and spatial proprioception (Wagle Shukla et al., 2013). SAI and proprioception were first normalized after 6 months, but not after 1 month of DBS. This study underscores the importance of chronic stimulation in the modulation of sensorimotor integration and proprioception.

The authors considered two possible mechanisms underlying SAI modulation by STN DBS. High-frequency DBS of the STN might normalize synchronization between basal ganglia structures, which might restore the ability of thalamocortical relay cells to respond to depolarizing inputs involved in sensorimotor integration (Brown et al., 2001; Rubin and Terman, 2004). Alternatively, STN DBS might have a direct effect on cortical structures through antidromic stimulation of the cortico-subthalamic pathway. In addition, the delayed effect of STN DBS on SAI may reflect long-term plastic changes in the sensorimotor cortex (Udupa et al., 2016). Whatever the underlying mechanisms may be, the modulatory effects of STN-DBS on SAI corroborate a sensorimotor integrative function of the STN as suggested by animal studies. Many STN neurons in the monkey (Wichmann et al., 1994) and patients with PD (Hutchison et al., 1998; Rodriguez-Oroz et al., 2001; Theodosopoulos et al., 2003) respond to cutaneous stimuli and passive movements. Alteration of sensory properties of the STN has been observed in animal models of PD. Peripheral sensory stimulation by hind paw pinch led to a greater increase in STN activity in dopamine-depleted rats than controls, suggesting altered STN sensitivity to afferent sensory inputs in the parkinsonian state (Magill et al., 2001).

## Is SAI Abnormal in PD?

To answer this question, we conducted a literature search on Pubmed^1^ using the following search strings: “Short afferent inhibition” OR “SAI” AND “PD.”

Exclusion criteria were as follows:

i. review articles or letter to the editors reporting no original data.
ii. studies about atypical parkinsonism [i.e., Progressive Supranuclear Palsy (PSP), Multisystemic Atrophy or Cortical Basal Syndrome], or dystonia not including PD population as control group.

This search resulted in 22 studies on the final search on November 2, 2018 (Tables s 1, 2). Fourteen studies reported a reduction of SAI in patients with PD. The average disease duration was 6.14 ± 4.39 years, mean ON UPDRS-III score was 24.03 ± 13.11, and the mean L-dopa equivalent dose 667.02 ± 314.51 mg across all positive studies (Table 1). In the remaining eight studies, six reported normal (Degardin et al., 2012; Zamir et al., 2012; Picillo et al., 2015; Dubbioso et al., 2017a; Ponzo et al., 2017; Nelson et al., 2018) and two found an enhanced SAI in PD patients (Di Lazzaro et al., 2004; Nardone et al., 2005). Mean disease duration was 5.63 ± 2.69 years, mean ON UPDRS-III score was 18.32 ± 8.52 and L-dopa equivalent dose was 633.94 ± 206.30 mg across all negative studies (Table 2).

**Table 1:** Studies showing short-latency afferent inhibition (SAI) alteration in Parkinson’s Disease (PD) patients.

Reference	Participants (mean age ± SD, y)	ON/OFF medication (UPDRS III)	Disease duration (years) or (months)°	L-dopa equivalent dose (mg)	Cognitive decline	SAI	Main findings	Interpretation
						ISI		
Versace et al. (2017)	15 PD borderline OERP (69.9 ± 5.4) 13 PD absent OERP (71.8 ± 5.4) 30 HC (67.4 ± 4.8)	PD-ON borderline OERP (16.1 ± 5.4) PD-ON absent OERP (16.9 ± 5.1)	PD-ON borderline OERP (7.7 ± 4.7) PD-ON absent OERP (8.0 ± 4.7)	PD-ON borderline OERP (516.7 ± 209.3) PD-ON absent OERP (530.8 ± 184.3)	MCI Level I in PD with absent OERP (92%) and in PD with borderline OERP (20%)*	N20+2 to +8 ms (1 ms Δ)	↓ SAI in PD with absent OERP	Altered SAI in PD patients with olfactory dysfunction and cognitive impairment
Oh et al. (2017)	28 PD hyposmia (63.04 ± 1.73) 26 PD anosmia (70.5 ± 1.88) 17 PD normosmia (67.35 ± 2.22) 20 HC (68.45 ± 1.61)	PD-ON hyposmia (18.96 ± 1.31) PD-ON anosmia (21.67 ± 2.27) PD-ON normosmia (14.94 ± 2.3)	PD-ON hyposmia (18.43 ± 1.56)° PD-ON anosmia (21.62 ± 1.68)° PD-ON normosmia (15.24 ± 1.78)°	PD-ON hyposmia (558.83 ± 47.02) PD-ON anosmia (544.7 ± 43.98) PD-ON normosmia (371.18 ± 43.8)	Normal MMSE	N20 to +4 ms (2 ms Δ)	↓ SAI in PD hyposmia and anosmia	Altered SAI in PD patients with olfactory dysfunction
Pelosin et al. (2016)	33 PD-fallers (7.6 ± 4.4) 17 elderly men-fallers (73.4 ± 4.2) 10 elderly men non-fallers (72.1 ± 4.9)	PD-ON (30.3 ± 9.13)	NA	775.5 ± 358.6	MCI Level I in PD-fallers*	N20-2 to +8 ms (2 ms Δ)	↓ SAI in PD-fallers and elderly men fallers	Altered cholinergic activity in patients with gait disturbances
Lee et al. (2015)	12 PD with dysphagia (73.33 ± 6.48) 17 PD without dysphagia (71.06 ± 6.98) 11 HC (62.4 ± 6.2)	PD-ON with dysphagia (24.17 ± 6.53) PD-ON without dysphagia (20.06 ± 4.07)	PD-ON with dysphagia (17.92 ± 9.16)° PD-ON without dysphagia (11.94 ± 4.98)°	PD-ON with dysphagia (589.58 ± 254.50) PD-ON without dysphagia (488.82 ± 181.86)	Normal MMSE	N20 to +4 ms (1 ms Δ)	↓ SAI in PD with dysphagia	Altered cholinergic activity in patients with dysphagia
Wagle Shukla et al. (2013)	11 PD with STN-DBS (58 ± 8.3) 10 HC (56 ± 7.1)	12 CONDITIONS	13.18 ± 4.21	1576.09 ± 1076.22	NA	N20	SAI is normalized 6 months after STN-DBS implant	Long-term STN-DBS improves sensorimotor integration and proprioception
Brusa et al. (2014)	10 PSP (59.3 ± 6.6) 10 PD (58 ± 6.4) 10 HC (57.2 ± 6.2)	PSP-ON (62.6 ± 9.4) PD-ON (36.2 ± 9)	PSP-ON (7 ± 1.2) PD-ON (6.3 ± 1.5)	PSP-ON (500 ± 150) PD-ON (750 ± 125)	Normal MMSE	N20-4 to +8 ms (4 ms Δ)	↓ SAI in PSP compared to PD, no effect induced by cerebellar iTBS	Cerebellar iTBS modulates cerebellar-cortical connectivity without affecting sensorimotor integration in PSP
Celebi et al. (2014)	11 MSA-P (58.7 ± 2.6) 8 MSA-C (58.9 ± 2.1) 10 PD (61.8 ± 1.8) 10 HC (65.8 ± 2.1)	PD-ON (8.8 ± 1.6)	3.2 ± 0.6	545 ± 57.6	MCI Level I in MSA type C and P*	N20+1 to +4 ms (1 ms Δ)	↓ SAI in MSA-C compared to PD and correlates with neuropsychological scores	Cholinergic dysfunction in MSA-C patients and cognitive impairment
Yarnall et al. (2013)	11 PD with MCI (73.3) 11 PD without MCI (66.9) 22 HC (67.9)	PD-ON with MCI (28.5) PD-ON without MCI (28.7)	PD-ON with MCI (20)° PD-ON without MCI (28.5)°	PD-ON with MCI (343.5) PD-ON without MCI (288.1)	MCI Level I in PD*	N20	↓ SAI in PD with MCI and correlates with MoCA score	Cholinergic dysfunction occurs early in PD with MCI
Nardone et al. (2013)	10 PD with RBD (65.9 ± 6.5) 13 PD without RBD (63.7 ± 6.4) 15 HC (66.4 ± 7)	PD-ON with RBD (17.5 ± 4.3) PD-ON without RBD (18.3 ± 4.3)	PD-ON with RBD (5 ± 2.3) PD-ON without RBD (6 ± 0.8)	PD-ON with RBD (578 ± 2.9) PD-ON without RBD (627 ± 341)	MCI in PD with RBD (90%) and without RBD (38%)^#^	N20+2 to +8 ms (1 ms Δ)	↓ SAI in PD with RBD and correlates with neuropsychological scores	Cholinergic dysfunction in PD with RBD and cognitive impairment
Rochester et al. (2012)	22 PD (70.18 ± 9.67) 22 HC (67.43 ± 8.43)	PD-ON (29.14 ± 9.54)	19.83 ± 8.6°	304.86 ± 130.91	MCI Level I in PD*	N20 to +4 ms (1 ms Δ)	↓ SAI in PD	Altered cholinergic activity in PD correlates with gait dysfunction
Celebi et al. (2012)	10 PD (72 ± 1.4) 10 PD with dementia (75 ± 2.2) 10 AD (76 ± 1.7) 10 HC (72.1 ± 2.3)	PD-ON (9.8 ± 1.6) PD-ON with dementia (22.9 ± 1.7)	PD-ON (2.3 ± 0.5) PD-ON with dementia (8.4 ± 1.6)	PD-ON (345.5 ± 77.7) PD-ON with dementia (943 ± 147.2)	Mild-moderate dementia	N20+1 to +8 ms (1 ms Δ)	↓ SAI in PD with dementia and AD	Cholinergic dysfunction in PD with dementia
Manganelli et al. (2009)	10 PD with VH (70.4 ± 5.3) 12 PD without VH (65.5 ± 10.1) 11 HC (62.4 ± 6.2)	PD-ON with VH (70.4 ± 5.3) PD-ON without VH (65.5 ± 10.1)	PD-ON with visual hallucination (8.7 ± 6.3) PD-ON without visual hallucination (9 ± 5.5)	PD-ON with visual hallucination (535.9 ± 307.8) PD-ON without visual hallucination (697.9 ± 271.5)	MCI in PD with VH (90%) and without VH (58%)^§^	N20+2 to +8 ms (2 ms Δ)	↓ SAI in PD with VH	Cholinergic dysfunction in PD patients with visual hallucinations and cognitive impairment
Sailer et al. (2007)	7 PD with STN-DBS (56.1 ± 3.6) 7 HC (56 ± 6.3)	PD-ON-STIM-OFF (10.86 ± 3.44) PD-ON-STIM-ON (7 ± 2.38) PD-OFF-STIM-OFF (6.17 ± 2.14) PD-OFF-STIM-ON (5.17 ± 2.23)	14 ± 4.51	723.14 ± 412.62	NA	N20+3	↓ SAI in PD-ON stimulator-OFF, SAI is restored by stimulator-ON	STN-DBS improves acutely sensorimotor integration
Sailer et al. (2003)	10 PD (58.2 ± 9.8) 10 HC (59.5 ± 10.7)	PD-ON (12.8 ± 6.3) PD-OFF (23.7 ± 11.1)	7.4 ± 5.7	835.25 ± 614.08	NA	N20 (MN) N23 (D3)	↓ SAI in PD-ON more affected side and normal in PD-OFF	SAI is altered by dopaminergic medication and may contribute to the side effects of dopaminergic drugs

MN, median nerve stimulation; D3, digit 3 stimulation; iTBS, intermittent theta-burst-stimulation; NA, not applicable; PD, Parkinson’s Disease; HC, healthy control; VH, visual hallucination; RBD, REM-sleep Behavior Disorders; MSA-P, multiple system atrophy parkinsonian type; MSA-C, multiple system atrophy cerebellar type; PSP, progressive supranuclear palsy; AD, Alzheimer disease; OERP, Olfactory Event-Related Potentials; STN-DBS, subthalamic nucleus deep brain stimulation; MMSE, mini mental state examination; MoCA, Montreal Cognitive Assessment; MCI, mild cognitive impairment; *MCI criteria according to the Level I of the MDS (Movement Disorder Society) commissioned Task Force (Litvan et al., 2012); ^#^MCI criteria according to Petersen and Morris, 2005; ^§^MCI criteria according to Caviness et al., 2007.

**Table 2:** Studies showing normal or enhanced SAI in PD patients.

Reference	Participants (mean age ± SD, y)	ON/OFF medication (UPDRS III)	Disease duration (years) or (months)°	L-dopa equivalent dose (mg)	Cognitive decline	SAI	Main findings	Interpretation
						ISI		
Nelson et al. (2018)	10 PD (61 ± 8) 11 HC (52.3 ± 10.4)	ON (12.2 ± 9.8) OFF (19.8 ± 7.9)	5.9 ± 3.42	614.2 ± 179	NA	N20+3	Normal SAI in ON and OFF in both side	PD demonstrates reduced activation of S1 (fMRI) and long sensorimotor integration (LAI)
Dubbioso et al. (2017a)	11 PD (62.3 ± 2.2) 8 LRRK2-PD (61.96 ± 3.9) 10 HC (59.4 ± 1.6)	PD-ON (23.4 ± 4.7) LRRK2-PD-ON (19.8 ± 7.9)	PD-ON (8.6 ± 1.2) LRRK2-PD-ON (8.4 ± 2.1)	PD-ON (838.1 ± 117.1) LRRK2-PD-ON (1015.9 ± 231.2)	NA	N20+2 to +8 ms (2 ms Δ)	Normal SAI	LRRK2-PD exhibits normal fast sensorimotor integration, but reduced motor cortex plasticity
Ponzo et al. (2017)	10 PD (65.7 ± 9,8) 8 LRRK2-PD (64.2 ± 8.3) 10 HC (63.5 ± 4.0)	PD-ON (9.5 ± 2.7) PD-OFF (24.2 ± 2.9) LRRK2-PD-ON (11.5 ± 4.2) LRRK2-PD-OFF (26.0 ± 9.3)	PD (7.4 ± 4.2) LRRK2-PD (8.5 ± 5)	PD (620 ± 87.1) LRRK2-PD (557.8 ± 53.4)	NA	N20-4 to +8 ms (4 ms Δ)	Normal SAI	LRRK2-PD exhibits normal fast sensorimotor integration, but increased motor cortex plasticity and disinhibition.
Picillo et al. (2015)	14 PD with FOG (63 ± 12) 10 PD without FOG (65 ± 10) 11 HC (62.4 ± 6.2)	PD-ON with FOG (17 ± 12) PD-ON without FOG (13.5 ± 8)	PD-ON with FOG (6.5 ± 4) PD-ON without FOG (5 ± 2)	PD-ON with FOG (1022.5 ± 771.2) PD-ON without FOG (560 ± 255)	MCI Level I in PD with FOG (71.4%) and without FOG (10%)*	N20+2 to +8 ms (2 ms Δ)	Normal SAI	Normal SAI in PD patients with FOG
Zamir et al. (2012)	12 PD (64.7 ± 10.3) 10 HC (63.1 ± 8.8)	PD-ON (13.6 ± 5.1) PD-OFF (23.1 ± 9.1)	7.3 ± 3.2	767.9 ± 484.3	Normal	N20+3	SAI is not modulated by iTBS	iTBS produces similar effects on cortical excitability for PD and controls
Degardin et al. (2012)	11 PD ON (61.5 ± 8.5) 8 PD OFF (61.3 ± 9.6) 10 PD *de novo* (60.6 ± 11.8) 11 PD ON-sham (61.5 ± 9.9)	PD-ON (17.4 ± 8.1) PD-OFF (29.3 ± 8.6) PD *de novo* (13.6 ± 4.5) PD-ON sham (19.5 ± 11.6)	PD-ON (6.8 ± 2.7) PD-OFF (6.2 ± 2.5) PD *de novo* (1.8 ± 1) PD-ON sham (8.2 ± 5.2)	PD-ON (785 ± 418) PD-OFF (646 ± 282) PD *de novo* (0) PD-ON sham (754 ± 485)	NA	N20	SAI is not modulated by iTBS	iTBS might improve both akinesia and sensory processing in patients with PD taking levodopa
Nardone et al. (2005)	8 PSP (68.2 ± 10.5) 10 PD (66.5 ± 11.8) 15 HC (NA)	PSP-OFF (36.4 ± 24.8) PD-OFF (39.7 ± 17.2)	8 PSP (22.4 ± 10.5)° 10 PD (19.1 ± 9.2)° 15 HC (NA)	8 PSP (68.2 ± 10.5) 10 PD (66.5 ± 11.8) 15 HC (NA)	Dementia in 60% of PD and in 50% of PSP (DSM-III criteria)	N20+2 to +8 ms (1 ms Δ)	↑ SAI in PD Normal SAI in PSP and HC	Different cholinergic dysfunction between PSP and PD
Di Lazzaro et al. (2004)	3 PD *de novo* (67.3 ± 9.1) 12 HC (73.1 ± 5.4)	PD-OFF affected side (8.7 ± 4.6)	NA	NA	NA	N20+2 to +8 ms (1 ms Δ)	↑ SAI more affected side compared to the less affected side and HC	SAI is enhanced in the more affected side in PD-OFF medication

iTBS, intermittent theta-burst-stimulation; NA, not applicable; PD, Parkinson’s Disease; HC, healthy control; FOG, freezing of gait; PSP, progressive supranuclear palsy; MMSE, mini mental state examination; MoCA, Montreal Cognitive Assessment; MCI, mild cognitive impairment; *MCI criteria according to the Level I of the MDS (Movement Disorder Society) commissioned Task Force (Litvan et al., 2012).

Importantly, these studies found consistent reduction of SAI mainly in medicated PD patients, whereas the off state was not associated with SAI alterations. The idea that nigrostriatal dopaminergic denervation does not reduce SAI or might even enhance cortical inhibition is supported by two studies. The first one, a small study on three drug-free patients with pure hemiparkinsonism (Di Lazzaro et al., 2004) showed enhanced SAI on the affected side. The second one, performed on 10 PD patients in off-state confirmed the increased cortical inhibition respect to patients with PSP and healthy controls (Nardone et al., 2005). The enhancement of SAI in the affected side might be related to an increase of cholinergic muscarinic activity in the contralateral cerebral cortex. Altered muscarinic cortical activity in PD is also supported by several post-mortem studies that have shown an increase in the total number of muscarinic cholinergic receptors in the cerebral cortex (Ruberg et al., 1982; Sirviö et al., 1989; Lange et al., 1993).

An intriguing and alternative hypothesis might be that a reduced thalamo-cortical drive caused by nigrostriatal dopaminergic denervation may increase SAI, an effect which might be obscured by chronic dopamine replacing therapy.

In 2003, Sailer et al. (2003) systematically examined the effect of dopaminergic therapy in 10 PD patients on and off medication. Patients only showed a reduction in SAI when they were on medication, and the medication-induced reduction in SAI only emerged on the more affected side. The medication-related SAI reduction can be restored acutely with STN-DBS (Sailer et al., 2007). This finding was largely confirmed by a recent meta-analysis which only found a consistent reduction in SAI across studies for PD patients on medication, but the attenuating effect of medication on SAI in PD was retrieved in the meta-analysis regardless of the affected side (Martin-Rodriguez and Mir, 2018). Moreover, the meta-analysis revealed an association between SAI changes and disease severity as well as cognitive deficits. Specifically, SAI impairment scaled with cognitive deficits in the four major cognitive domains, although the strongest association was found for visuospatial and executive deficits.

**Figure 1:**
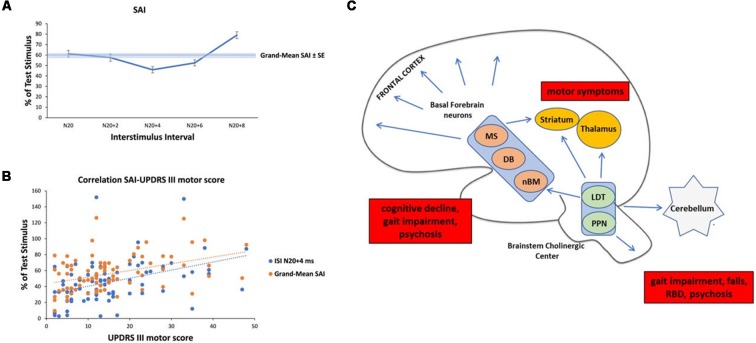
Analysis of short-latency afferent inhibition (SAI) in our Parkinson’s Disease (PD) cohort and schematic representation of cholinergic sources in the human brain with their clinical correlates in PD. **(A)** Temporal evolution of SAI in our cohort of PD patients. The horizontal axis shows inter-stimulus interval (ISI) values (the time between the peripheral stimulation and cortical stimulation). ISIs were determined by adding 0, 2, 4, 6, and 8 ms to the latency of the N20 component. The vertical axis shows the percentage of test motor evoked potential (MEP) at each ISI. **(B)** Linear positive correlation between SAI at ISI N20+4 ms, Grand-Mean SAI and UPDRS III motor score in medicated patients. **(C)** Schematic representation of the three major sources of cholinergic projections in the brain and main clinical correlates in PD (red boxes). Basal forebrain neurons, including the nucleus basalis of Meynert (nbM), medial septal nucleus (MS) and diagonal band of Broca (DB) provide the cholinergic projections to the cerebral cortex and are responsible for cognitive impairment, gait impairment and psychosis. The pedunculopontine nucleus-laterodorsal tegmental complex [referred to as the pedunculopontine tegmental nucleus (PPN) and LDT], a brainstem center, provides cholinergic inputs primarily to the thalamus, but also has connections to the cerebellum, several brainstem nuclei, some striatal fibers, and the spinal cord. This system is mainly involved in walking disturbances, rem-sleep behavior disorders (RBDs) and psychosis. In addition, small populations of intrinsic cholinergic neurons are present in the hippocampus, striatum (cholinergic interneurons), parts of the reticular formation, and cerebellum. The cholinergic interneurons might be the main cause of motor symptoms in PD.

Prompted by these findings, we pooled SAI data from our database and three previous studies (Manganelli et al., 2009; Picillo et al., 2015; Dubbioso et al., 2017a). The pooled data set included measurements from 81 PD patients (57 men) with a mean age of 64.37 ± 7.57 years, average disease duration of 8.41 ± 4.61 years, mean ON UPDRS-III score of 15.48 ± 10.89, and a daily L-dopa equivalent dose of 833.55 ± 470.70 mg. SAI was tested in all patient on the more affected side while they were taking their normal medication. SAI measurements covered five interstimulus intervals adjusted to the individual N20 wave latency (N20+0 ms, N20+2 ms, N20+4 ms, N20+6 ms, N20+8 ms). Patient’s UPDRS III motor score in the ON medication state was the only variable that showed a positive linear correlation with SAI at an ISI of N20+4 ms (*ρ* = 0.405; *p* < 0.01) and with the mean SAI across all five interstimulus intervals (*ρ* = 0.401; *p* < 0.01, Figures 1A,B). Indeed, neither disease duration nor daily dopaminergic medication showed a significant relationship with SAI (all *p* ≥ 0.105). Overall, the results suggest that the attenuating effect of medication state on SAI is more pronounced in patients in whom dopamine replacement therapy shows limited efficacy to normalize parkinsonian motor symptoms as indicated by high UPDRS scores in the on-medication state. It is conceivable that patients who show a less favorable response to dopamine replacement therapy may also have more cholinergic deficits and hence the reduction in SAI may, at least in part, resulting from a co-existing cholinergic deficit at the cortical level. Interestingly, patients with atypical parkinsonism (i.e., Progressive Supranuclear Palsy or Multisystemic Atrophy) that usually respond insufficiently to dopaminergic medication, might exhibit reduced levels of SAI (Brusa et al., 2014; Celebi et al., 2014).

## Does SAI in PD Scale With Gait Problems and Non-motor Symptoms?

PD causes a wide range of non-motor symptoms which may even precede the manifestation of the classic motor symptoms (Riedel et al., 2010). Non-motor symptoms include cognitive dysfunctions and decline, apathy, psychiatric disturbances (depression, psychosis, impulse control), autonomic failure (gastrointestinal, cardiovascular, urinary, sexual ability, thermoregulation), sleep disorders, and pain syndrome (Chaudhuri and Schapira, 2009). In recent years, SAI has been increasingly used in PD to identify whether specific **motor and non-motor symptoms** scale with abnormalities in SAI, presumably due to cholinergic and dopaminergic cortical dysfunction.

KEY CONCEPT 4 **Motor and non-motor symptoms**

Sources of considerable burden in people with PD are the typical motor symptoms, such as resting tremor, rigidity, bradykinesia, postural instability and non-motor symptoms, namely cognitive declines, psychiatric disturbances, autonomic failures, sleep difficulties, and pain syndrome. These symptoms are variously associated with dopaminergic/cholinergic neurodegeneration and SAI alteration at the cortical level.

Among motor disturbances, gait abnormalities and falls have attracted attention for their association with cognitive decline and cholinergic dysfunction in PD (Newman et al., 2012; Perez-Lloret and Barrantes, 2016). Indeed, reduced SAI has been proven to be an independent predictor of slower gait speed (Rochester et al., 2012) and associated with a higher falls risk in PD (Pelosin et al., 2016). However, in a recent study performed on PD with freezing of gait (FOG; Picillo et al., 2015) the authors failed to prove alteration of SAI in this subtype of patients. Since gait disturbances in PD are heterogeneous and may be underpinned by different neurotransmitters and circuits, it is to be expected that the relation between SAI and gait deficits may be complex and the modulatory role of medication state also needs to be factored in when addressing this issue.

Regarding non-motor symptoms, SAI abnormalities have been found associated with dementia and Mild Cognitive Impairment (MCI; Celebi et al., 2012; Yarnall et al., 2013), confirming the role of cholinergic dysfunction in the development of cognitive impairment in PD. Indeed, SAI has been found to be reduced in PD patients with those symptoms associated with a higher risk of cognitive decline, such as visual hallucinations (VH; Manganelli et al., 2009), dysphagia (Lee et al., 2015), olfactory dysfunction (Oh et al., 2017; Versace et al., 2017) and REM-sleep Behavior Disorders (RBDs; Nardone et al., 2013). These studies are in agreement with the idea that the cholinergic dysfunction makes a major contribution to non-motor symptoms and associated cognitive deficits in PD (Marra et al., 2012; Newman et al., 2012).

A recent review summarized central cholinergic sources of the healthy human brain in two main tracks (Newman et al., 2012). On the one hand, brainstem nuclei, including the pedunculopontine tegmental nucleus (PPN) and the laterodorsal pontine tegmentum, send cholinergic projections to the thalamus, basal ganglia, basal forebrain and to a much lesser extent, the cerebral cortex. On the other hand, the magnocellular basal forebrain-cholinergic systems, including the nucleus basalis magnocellularis and nucleus basalis of Meinert (NBM) send major projections to neocortex, entorhinal cortex, limbic cortices, cingulate cortex, and hippocampus. In addition, small populations of intrinsic cholinergic neurons are present in the hippocampus, striatum (cholinergic interneurons), parts of the reticular formation, and cerebellum (Bohnen and Albin, 2011; Manganelli et al., 2013; Dubbioso et al., 2015). These cholinergic nuclei and their projections have selectively degenerated in PD (Bohnen and Albin, 2011; Perez-Lloret and Barrantes, 2016). Thus, we speculate that SAI in PD may mainly reflect a cortical cholinergic deficit due to cholinergic neurodegeneration. The cortical cholinergic imbalance may derive from many sources that are variably impaired according to disease severity and symptoms. For example, degeneration of cholinergic striatal tone is responsible for motor symptoms, alteration of the NBM and/or PPN nuclei for gait impairment and falls, cognitive decline, RBD, psychosis (Perez-Lloret and Barrantes, 2016), see Figure 1C.

## Conclusion and Outlook

In this review, we have discussed the possible contributions of the fast-afferent somatosensory pathway, the intracortical integrative component and the fast-efferent corticomotor pathway to alterations of SAI in PD. We concluded that PD-related changes in SAI are most likely caused at the cortical level, where sensory input is rapidly integrated into a motor output. This makes SAI a useful tool to probe how PD impacts on the sensorimotor integration processing at the cortical level.

Studies performed on PD patients have shown variable results, ranging from reduced to normal or even enhanced SAI findings. Several factors may be responsible for these heterogenous results such as between-group differences in disease severity, disease duration, dopamine replacement therapy and cognitive status. While patients with PD show normal levels of SAI in the off-medication state, SAI is reduced in the on-medication state, suggesting a role of dopamine replacement in driving this abnormality. Interestingly, previous research has suggested that dopaminergic medication could lead to decreases in central processing or integration of sensory signals in PD patients. For instance, it has been shown that dopaminergic medication could worsen SAI and proprioception (distal and spatial errors) and were normalized by chronic STN-DBS, likely through long-term plastic changes in the basal ganglia thalamocortical circuit (Wagle Shukla et al., 2013). Yet, pharmacological studies which systematically study dose-dependent effects of dopamine replacement therapy on SAI magnitude in PD are still lacking. The positive relationship between residual parkinsonian motor symptoms and SAI suppression in the on-medication state suggests that the effect of dopamine replacement may be more detrimental to SAI in patients that are less responsive to dopaminergic pharmacotherapy, for instance, patients with more prominent cholinergic involvement. This would also explain why non-motor symptoms have been associated with a reduction of SAI in PD.

Some important aspects of SAI still remain to be explored in PD. For instance, by systematically varying the intensity of peripheral stimulation one may derive a stimulus-response curve of SAI that may be more sensitive to intrinsic disease-related but also therapy-related changes in SAI. Furthermore, the application of homotopic or heterotopic somatosensory stimulation may reveal interesting insights into the altered center-surround organization of fast sensorimotor integration at the cortical level (Dubbioso et al., 2017c).

Future research on SAI in PD should focus on validating SAI as a biomarker of central cholinergic activity through a multimodal approach by combining neurophysiological results with neuroimaging. For example, correlation analysis with structural data (i.e., analysis of gray matter volume, diffusion tensor imaging) of the main cholinergic system nuclei, would reveal a structure-function relationship between SAI changes and structural cholinergic denervation. The introduction of new PET radioligands, such as (18 F) fluoroethoxybenzoyesamicol [(18 F) FEOBV], a ligand which shows a high affinity for the vesicular acetylcholine transporter, will enable the researcher to simultaneously examine functional changes of the cholinergic system* in vivo*. This line of research will help to clarify the role of impaired cholinergic neurotransmission in the development of motor and non-motor symptoms in PD.

## Author Contributions

All authors prepared the manuscript draft and approved the final manuscript.

## Conflict of Interest Statement

HS has received honoraria as speaker from Sanofi Genzyme, Denmark and Novartis, Denmark, as consultant from Sanofi Genzyme, Denmark and as senior editor (NeuroImage) from Elsevier Publishers, Amsterdam, Netherlands. He has received royalties as book editor from Springer Publishers, Stuttgart, Germany. The remaining authors declare that the research was conducted in the absence of any commercial or financial relationships that could be construed as a potential conflict of interest.

## Footnotes

^1^https://www.ncbi.nlm.nih.gov/pubmed/
